# Lithuanian polyphonic songs *sutartinės*: the archaic nature of their musical language in the context of global music

**DOI:** 10.3389/fpsyg.2024.1285394

**Published:** 2024-02-22

**Authors:** Eirimas Velička

**Affiliations:** Rhythmic Music Department, Faculty of Arts and Creative Technologies, Vilnius University of Applied Sciences, Vilnius, Lithuania

**Keywords:** *sutartinės*, ethnomusicology, archaic music, traditional songs, world music, musical language, vocal polyphony, polyrhythm

## Abstract

Lithuanian traditional polyphonic songs, known as *sutartinės*, are characterized by a distinctive musical language and have almost no analogues in world music. The aim of this article is to explore the peculiarities of their musical language and the socio-cultural context of their performance tradition in order to reveal their archaic origins. The archaic nature of *sutartinės* songs is shown not by individual features of their musical language, but by the totality of these features, the peculiarities of their poetics, and performance traditions. An examination of the musical elements and poetry of these songs, and their juxtaposition against examples of archaic vocal polyphony from other cultures, leads to the convergence of arguments in favour of the very ancient origins of these songs, possibly dating back to Old Europe (c. 3 millennium BC). A deeper insight into *sutartinės* songs significantly enriches our understanding of the origin and development of traditional vocal polyphony.

## 1 Introduction

Lithuanian traditional polyphonic songs, known as *sutartinės*, enjoy a distinctive and significant place in the world’s musical heritage. These songs are characterised by a specific musical language, archaic texts and elements of ritual choreography. Most of the *sutartinės* songs have the following features: (1) linear polyphony; intertwining voices with regular or frequent harmonization at the interval of second; (2) narrow melodic range and limited number of scale steps; (3) polyrhythms and rhythmic complementarity with frequent syncopation; (4) two different texts performed simultaneously; (5) stanzaic structure, where a stanza consists of a meaningful text and a constantly recurring refrain of asemantic words or syllables; (6) the syncretic nature of the performance, where music, text and movement are closely linked.

In Lithuania, alongside with vocal polyphonic songs, there are also instrumental *sutartinės*. They are performed on traditional instruments: the five-stringed psaltery (known in Lithuanian as *kanklės*), the separate pan pipes (*skudučiai*), and the long-necked wooden trumpets (*daudytės*). Instrumental *sutartinės* will not be covered in this article, mentioning only that the songs performed on wooden horns (*ragai*) and separate pipes (*skudučiai*) are based on the complementary polyrhythms and harmony of seconds between adjacent voices, giving the impression of a rhythmically moving cluster (for example, LLIM; Paliulis, 1959, 97).^1^ The closest analogues of these instrumental pieces in world music are the Solomon Islands Pan Flute Ensembles and the instrumental wind music of Baka and other pygmy groups in Central Africa. As Lithuanian wind instruments were made of nondurable natural materials (such as birch bark, angelica sylvestris grass stems), there are no surviving archaeological examples of them. We can therefore only speculate about the age of these instruments based on their analogues in other parts of the world. Remote analogues of Lithuanian instruments in Central Africa and Melanesia suggest that the age of these instruments and the music they play dates back to the Stone Age. However, this approach should not be over-absolutized. Given the complexity of Lithuanian and Central African instrumental polyphonic music (*cf.* LLIM; Paliulis, 1959, see also Arom, 1991), it is possible that this polyphony is the result of a long-term evolution.

Vocal and instrumental *sutartinės* survive in a delimited territory of Lithuania (the north-eastern part of the country), and form a completely separate genre of musical folklore. *Sutartinės* have almost no counterparts in the world; those musical traditions that share similarities are geographically and culturally quite distant. Due to their uniqueness, *sutartinės* have attracted great interest amongst ethnomusicologists not only in Lithuania but also internationally. Of great interest are the archaic forms of polyphony, polyrhythmics, and second interval harmony between the voices of *sutartinės* songs. The uniqueness of this genre, its distinctive musical and poetic language, the syncretism of music, word and movement, and the social context of their performance allow us to speculate on the archaic nature of this genre of traditional polyphony, which may date back to the times of Old Europe. Structural and comparative research on the musical language of these songs could give us a clearer understanding of the evolutionary aspects of musical thinking and the genesis of musical language.

The aim of this article is to analyse the musical language of *sutartinės*, to discuss the peculiarities of their performance and the socio-cultural context of their existence. Through analysis of their musical language, structure and lyrics, and by comparing them with examples of vocal polyphony from other cultures, their archaic origins, dating back to the times of Old Europe, is discussed.

## 2 An overview of the research on the *sutartinės*, and their present-day viability

The first mention of *sutartinės* songs in written sources is found in a 16th century historical chronicle (Stryjkowski, 1582, p. 85–86, 146). The text mentions the characteristic refrain *łado* in these songs and the singing ‘one another mouth to mouth’. The author attributes the text of the song quoted in the Chronicle to a historical event—the burning of Kaunas castle by the Crusaders in 1362 (Landsbergis, 1993, p. 39–40). Other, later sources mention long wooden trumpets, men and women playing and singing alternately (Guagnini, 1578, p. 129; Lepner, 1744, p. 96; Bretkūnas, 1983). The first notes and publications of *sutartinės* began only at the beginning of the 19th century, when their living tradition had already begun to disappear (Daukantas, 1845; Neselman, 1853; Stanevičius, 1954).

At the beginning of the 20th century, more consistent scientific research on *sutartinės* arrangements began. One of the first Lithuanian researchers, A. Sabaliauskas (Sabaliauskas, 1911), together with the Finnish scholar A. R. Niemi, compiled and published the first collection of songs to include s*utartinės* (Niemi and Sabaliauskas, 1912). Z. Slaviūnas has made a significant contribution to the research of *sutartinės* songs. He researched the types of polyphony, geographical distribution, and rhythmic characteristics of the songs (Slaviūnas, 2006), and published a three-volume set of 1820 *sutartinės* songs in 1958–1959. This publication is still the most important printed source for songs of this genre (SlS; Slaviūnas, 1958/1959).^2^ Instrumental songs were explored by Stasys Paliulis. He compiled a collection of *sutartinės* music for Lithuanian folk wind instruments (LLIM; Paliulis, 1959).^3^ Another 35 songs were published in the collection *Aukštaičių Melodijos* by L. Burkšaitienė (AM; Burkšaitienė and Krištopaitė, 1990). These are the main sources in which most of the surviving song samples are printed.

The most prominent contemporary researcher of *sutartinės*, D. Račiūnaitė-Vyčinienė, has published several dozen scientific articles and three monographs on *sutartinės*, one of them in English (Račiūnaitė-Vyčinienė, 2002). Her work analyses the origin and spread of *sutartinės*, their syncretism and performance peculiarities. The first monograph’s appendix publishes *sutartinės* songs written at the end of the 20th century and previously unpublished (Račiūnaitė-Vyčinienė, 2000). Her latest monograph is rich in comparative musicology, comparing *sutartinės* songs with examples of archaic vocal polyphony from the Ainu of Japan, the Inuit of Canada and the Balkans.

The work of other Lithuanian musicologists is also relevant to the study of *sutartinės*. J. Antanavičius revealed analogies between the polyphonic principles and forms of these songs and professional academic music (Antanavičius, 1969). R. Gaidamavičiūtė studied the rhythmic features of *sutartinės* (Gaidamavičiūtė, 1981). Ambrazevičius studied the psychoacoustic characteristics of *sutartinės* performance and perception (Ambrazevičius, 2003). He found that the harmony of these songs does not empoy the exact intervals of tone and semitone, but rather approximate seconds (approx. 180 cents) that evoke a feeling of ‘maximum roughness’. Nakienė investigated the analogies between vocal and instrumental *sutartinės* (Nakienė, 2003), and the typological links between polyphonic and monophonic songs, putting forward a bold, yet unproven, hypothesis about the possible origin of *sutartinės* from monodic refrain songs (Nakienė, 2001).

Lithuanian polyphonic songs have been mentioned in fragments by ethnomusicologists from other countries (Wiora, 1952, p. 7; Sachs, 1962, p. 181; Emsheimer, 1964, p. 46; Danckert, 1966, p. 66; Messner, 1980; Elschekova, 1981; Brambats, 1983, p. 26). These draw attention to the distinctive polyphony of *sutartinės* songs, the intervals of seconds between the voices, and make comparisons them with analogous examples of vocal polyphony from other cultures. Sachs, for example, pointed out that the second intervals between the voices of *sutartinės* are not caused by the vertical harmonisation, but by the horizontal movement of the voices. ‘The case of second parallels must be dismissed from the realm of vertical hearing and dissonance. Otherwise, we could not find in a country as musical as Lithuania actual folksong Canons with consistent clashes of semitones and wholetones. Attention, strictly horizontal and focused on the re-entry of the theme in another voice every four measures, completely ignores the constant frictions on our vertically trained ears’ (Sachs, 1962, p. 181).

However, in the international context, fundamental works on *sutartinės* songs are scarce. Amongst the more significant works devoted to the study of this genre are the dissertation in German by Latvian musicologist M. Boiko (Boiko, 1992) and the monograph by D. Račiūnaitė-Vyčinienė in English (Račiūnaitė-Vyčinienė, 2002).

In the 1930s, when the tradition of *sutartinės* had already begun to decline, Lithuanian composers began to take an interest in them. With the formation of the Lithuanian national school of composition, quotations, motifs, and structural principles of the *sutartinės* appeared more and more frequently in the works of professional composers. J. Gruodis, S. Vainiūnas, B. Dvarionas, J. Juzeliūnas, B. Kutavičius and other composers used motifs from or quotations of *sutartinės* in their works. Juzeliunas’s Symphony No.5 for girls’ choir and string orchestra *Lygumų giesmės* (‘The Songs of the Plains’, 1982) is one of his most striking masterpieces, expanding the musical principles of *sutartinės* into a contrasting two-movement symphonic canvas.

The *sutartinės* proved to be particularly suitable for music pedagogy because of their structure and musical language, in line with Orff’s principle of elemental music. Since the 1960s, examples have been systematically used in Lithuanian mainstream schools and included in textbooks (Velička, 2017, p. 43, 211–221). In the 1970s and 1980s, due to the growing interest in folklore, the *sutartinės* experienced a revival. Many newly formed folklore ensembles have added them to their repertoire. In the 1990s, Lithuanian jazz, rock and electronic musicians and performers started to use *sutartinės* in their works and musical projects. It should be noted that the popularity of *sutartinės* is increasing amongst youth music groups and subcultural communities. In 2010, the Lithuanian polyphonic songs *sutartinės* were included in the UNESCO Representative List of the Intangible Cultural Heritage of Humanity.

## 3 The syncretism and characteristics of the performance of *sutartinės*

The main feature of the *sutartinės* songs is the collectiveness of their performance. The Lithuanian name of this genre emphasises this. The word ‘Sutartine’ is derived from the verb ‘sutarti’ (to agree, to be in concordance, to arrange), which has a double meaning: both ethical and aesthetic. Unlike most other traditional Lithuanian song genres, which are usually sung by a larger group of people, *sutartinės* are usually performed by two, three or four female singers. According to the number of performers and the way in which they are performed, *Sutartinės* are divided into three main groups: *twosomes*, *threesomes*, and *foursones*, each defining a distinct style according to the numer of performers. Researchers of *sutartinės* count from 19 to 38 different ways and variations of their performance (Slaviūnas, 2006, p. 36–124; Račiūnaitė-Vyčinienė, 2000, p. 86–144).

*Sutartinės* songs consist of two melodic lines and/or two lyrics performed simultaneously. The egalitarian nature of the performance of the songs must be emphasised: the vocal parts of these polyphonic songs are of equal importance. No vocal part is more or less important than another. Ethnomusicologists and philosophers of music have given examples of how the way music is performed reflects the social structure of a community (Blacking, 1973; Feld, 1984; Elliott, 1989). It is therefore reasonable to assume that Lithuanian polyphonic songs reflect a kind of community structure that is based not on vertical, hierarchical, but on horizontal interrelations. They reveal the communal nature of performance. Around one third of all published *sutartinės* are work songs (Slaviūnas, 2006, p. 125–133). Communality and mutual help in farm and field work remained a vibrant tradition in Lithuanian villages until the end of the 20th century.

Polyphonic songs are performed in such a way that two complementary voices are heard at the same time and interact with each other. Techniques such as paraphony, heterophony, antiphony and canon are used to interweave the voices in a way that creates a radically new sound quality. Polyphony is created, where the rhythms of the separate voices contrast or complement each other, often creating sharp dissonances of seconds between the voices. It should be noted that these intervallic seconds were not perceived as dissonances by the performers themselves. Paliulis, who has studied the *sutartinės*, says: ‘It must be assumed that our ancestors’ ears were fond of the interval of a second; it was apparently a consonance for them’ (Paliulis, 2002, p. 41). D. Račiūnaitė-Vyčinienė emphasises that *sutartinės* are not just an expression of harmonious sounding, but also of ‘universal harmony’ (Račiūnaitė-Vyčinienė, 2003, p. 136).

Intertwining voices are not perceived as two separate and independent melodic lines, but as a qualitatively new acoustic reality, where the totality of the sound is not a simple sum of individual melodic voices. The singers themselves used to compare the sound of the *sutartinės* to the ringing of a bell (Račiūnaitė-Vyčinienė, 2003, p. 139). Acoustic studies of *sutartinės* have revealed their similarities to the sound of bell (Ambrazevičius, 2003, p. 132). During the performance, the individual consciousness of the singers seems to merge and dissolve into the group consciousness, thus creating a new, higher-level collective entity. These polyphonic songs require a collective performance experience, which is gained by singing together for a certain period of time. Traditionally, therefore, the line-up of singers tended to remain almost unchanged: when one singer died in a village, the singing group was quite often disbanded.

*Sutartinės* were performed exclusively by women. The special status of singers in this genre highlights its archaic origins. As the tradition declined, female singers were sometimes referred to as fairies or witches (Račiūnaitė-Vyčinienė, 2018, p. 150). Lithuanian folklore is full of stories and fairy tales in which fairies or witches sing songs whilst washing laundry at night by a river or lake. In Lithuanian culture, the image of the witch is of very ancient origin and belongs to the old cultural layer of pre-Indo-European deities. The archaeologist M. Gimbutas, who has studied ancient European culture, considers the witch to be an inversion or degradation of the Great Goddess (Gimbutienė, 2002, p. 48, 55).

The musical material and performance of the *sutartinės* songs is based not on development but on constant repetition, emphasizing their cyclical nature. Their musical material is a clear embodiment and representation not of a linear but of a cyclical conception of time, such as the one described by Mircea Eliade in his book *Le Mythe de l’Eternal Retour* (Eliade, 1969). This feature also underlines their archaic origins.

Another distinctive feature of *sutartinės* is their syncretic quality. Scholars note that the phenomenon of these songs combines music, poetic text and ritual movement into one indivisible whole (Račiūnaitė-Vyčinienė, 2000, p. 40, Urbanavičienė, 2009, p. 17–30). In most of the *sutartinės* songs there is a very close relationship between music and movement. Some of the elementary movements that accompany the performance of *sutartinės* (limping step, bowing, etc.) can be interpreted as ‘proto-dance’. In many ancient cultures, music included not only sound but also ritualized movement—‘proto-dance’ (Blacking, 1995, p. 241). Elementary rhythmic movements help dancers to coordinate their actions, and can be traced back to the origins of musical rhythm, where synchronised movements reinforce social bonding. According to Dalia Urbanavičienė, of all the written and published *sutartinės*, approximately a sixth of them contain references to the choreography. Taking into account that some folklore collectors may not have marked the choreographic references, it can be assumed that the proportion of *sutartinės* performed with movements may have been much higher. The proportion of songs in other genres, performed with movement is much smaller.

Some elements of archaic choreography that accompany the performance of *sutartinės*, particularly highlight the links to archetypal figures or universal symbols. For example, the threesomes *sutartinės* are performed as a strict triple canon, which creates and embodies the archetypal image of the wheel in its sound. Many of the three-part *sutartinės* are performed whilst walking in a circle. Walking in a circle, simbolizing the movement of the sun, is typical of the ancient folklore fundations of many cultures (Račiūnaitė-Vyčinienė, 2000, p. 34). Particularly archaic is the ritual ‘bowing to the sun’, which each of the three singers performs in turn as they sing a three-part *sutartinė* about the sun (SlS; Slaviūnas, 1958/1959, 92, 93). This song is performed in the evening after the rye harvest, as the sun sets. Each verse repeats the refrain motif ‘Saulala sadina’ (*The sun is setting*).

Meanwhile, the foursomes *sutartinės* are performed by four female singers—two pairs of performers who take turns singing and often perform certain movements. For example, the singing pairs take two steps, either approaching each other and then retreating again or bowing to each other. Meanwhile, the other two stand still. This creates a cross, or a square shape, in the space. The walking of one couple in front of the other is considered to be one of the oldest forms of archaic choreography; Curt Sachs attributes them to the Neolithic period (Sachs, 1933, p. 107).

Some of the danced and played *sutartinės* contain relics of an animistic or totemic worldview. The main characters of their texts are a sparrow, a cuckoo or a goat. The ‘goat-hopping’ motif is particularly popular (SlS; Slaviūnas, 1958/1959, 1374–1387); songs about a goat abound in Lithuanian children’s songs (VD; Jokimaitienė and Puteikienė, 1980, 362–409; 760–766). The *sutartinė* about a sparrow is sung and danced (SlS; Slaviūnas, 1958/1959, 1367); a ‘limping step’ is performed during the refrain. The characters of the owl and the sparrow are often found in Lithuanian children’s songs (VD; Jokimaitienė and Puteikienė, 1980, 484–506, 542–574). In some of them, a sparrow invites an owl to dance and steps on its foot. Similar motifs are of very archaic origin.

Twosomes (two-part) *sutartinės* are usually performed without movement. However, scholars of these songs see links in their structure and performance with traditional woven and braided bands and their ornaments (Račiūnaitė-Vyčinienė, 2018, p. 14–17, 139–142). The voices of the performers seem to weave together, creating a sonic pattern or ornament. The terms used by the singers confirm this link. According to the singers, the text of the such a song is ‘assembled’ in a similar way to the way weaving patterns are made. This is particularly evident in the case of twosomes *sutartinės*, in which two interlocking voices in a musical fabric have a function analogous to that of warp and weft in textiles.

## 4 Texts of *sutartinės* songs

The strophes of *sutartinės* songs consist of a meaningful text and a refrain with no semantic meaning. Asemantic refrains sometimes make up 1/2 or even 3/4 of a strophe. There are also some songs whose entire text consists of an asemantic refrain. These are considered to be the original, most archaic form of *sutartinės*. The meaningful part of the text is in many cases related to the function of the song (Slaviūnas, 2006, p. 134). There are songs for work, calendar celebrations, weddings, christenings, and dance. The lyrics of the songs show their connection with various outdoor activities (hunting, hay harvest, rye harvest, oat harvest, yarn spinning, dung carting). The lyrics of *sutartinės* songs in calendar rites reflect the customs that were used to ensure good harvests and the success of work completed (sleigh rides at Carnival, swinging on swings at Easter, visiting the rye at Pentecost, the customs of the Midsummer festival). Mostly, there is no coherent narrative in the lyrics. The text is taught through constant repetition, with only one other word changed (e. g. ‘I sowed flax; flax sprouted, flax grew…’).

Amongst the more interesting texts, we should mention those that contain relics of the pre-Christian mythical worldview and traces of archaic rituals. The lyrics personalise animals and trees, and in some cases the Sun. In some songs, the Sun is planting a garden (SlS; Slaviūnas, 1958/1959, 600). The oak tree is compared to the father and the lime tree to the mother (SlS; Slaviūnas, 1958/1959, 23 a-b, 24, 28–30, 224–225, 65–67, 1721). The sister addresses the apple tree, which is golden and its apples are silver (SlS; Slaviūnas, 1958/1959, 635 a-b, 1362 a-b, 1363). The motif of ‘goat dancing’ is found in the group’s humorous songs (it is likely that they were ritual songs in the past). The goat is asked to show how the father, mother, brother, sister would dance (SlS; Slaviūnas, 1958/1959, 1374–1387). It should be noted that texts about the goat are especially abundant in Lithuanian children’s songs (VD; Jokimaitienė and Puteikienė, 1980, 362–429). Some of the lyrics depict individual parts of the (sacrificed?) goat’s body: eyes, ears, legs, tail (VD; Jokimaitienė and Puteikienė, 1980, 370). Other songs tell how a goat was eaten by wolves in the forest and only the horns were left. The horns were used by the birds of the forest to brew beer and make a big feast (VD; Jokimaitienė and Puteikienė, 1980, 381–392). These texts are thought to relate to the autumn rite of sacrificing a goat, traces of which can be found in written sources as early as the 13th century (Balsys, 2014; Vaiškūnas, 2014). The bees mentioned in numerous *sutartinės* lyrics (SlS; Slaviūnas, 1958/1959, 187, 304–319, 604 and others) are probably a legacy of ancient beekeeping. The motifs of the ‘Sparrow Hunt’ and the ‘Wedding of Birds’ are also quite archaic (SLS; Slaviūnas, 1958/1959, 1367, 1282–1284). These motifs are particularly abundant as well in children’s songs (VD; Jokimaitienė and Puteikienė, 1980, 633–647). We can only speculate from what times this poetic line might have come: *Pelėda pelėdėla, ųžuole tupėdama, giesmes taiso* (‘An owl, the little owl, perching in an oak tree, creates songs’: SLS; Slaviūnas, 1958/1959, 467, 468). Archaeologist Maria Gimbutas, who has studied ancient pre-Indo-European culture, argues that the Owl can be considered one of the most important attributes of the Great Goddess (Gimbutienė, 1985, p. 33, 34; Račiūnaitė-Vyčinienė, 2000, p. 66). It is likely that the beaked figurine from 5000–4500 BCE, found in Vinča (Transylvania), represents the Goddess-Bird in the form of an owl (Gimbutienė, 1996, p. 226). The symbol of the oak tree as the World Tree is also easily recognizable in this text. The texts of polyphonic songs contain rare words of old origin, archaic grammatical forms that are no longer used in the Lithuanian language today (e.g., *pa**mi**rodyk* = *parodyk* ***man*—**‘show to me’).

The most important part of these lyrics, however, is not the meaningful text itself, but the asemantic refrains—interjections made up of meaningless morphemes or words that have lost their original meaning. The interjectional refrains of the *sutartinės* are characterized by an incredible richness and variety of forms not found in songs of other genres (Sauka, 1970, p. 226–234). Such refrains usually make up 1/2 to 3/4 of the text, and in some cases the entire text of a song consists of only asemantic refrains (SlS; Slaviūnas, 1958/1959, 1556; 1587; 1667). Often the refrain is heard at the very beginning of the stanza, before the meaningful text. Although the words in the refrains have no syntactic meaning, some of them can be recognized. However, their grammatical forms are archaic, characteristic only in songs of this genre, and are no longer used in modern Lithuanian. Other interjection lexemes consists of nonsense words or syllables, sometimes of onomatopoeic origin. The refrain *Titity, tatato* clearly imitates the sound of a natural wood trumpet, called *daudytė* (SlS; Slaviūnas, 1958/1959, 482–484). The onomatopoeic origin of this refrain is underlined by the melodic line of the song, which is based on a ‘fanfare tune’ (it consists of the 4th and 5th harmonics of the harmonic series). The refrain *lioj lylia*, repeatedly performed in the context of a second paraphony, creates a sound imitating the voices of flying cranes (SlS; Slaviūnas, 1958/1959, 109a-b). Comparisons with the voices of cranes, the honking of and the clucking of hens are also mentioned by the performers of the songs themselves (Račiūnaitė-Vyčinienė, 2018, p. 394).

Due to the polyphony of the music and lyrics, the meaningful words of the *sutartinės* songs are quite difficult to hear and understand because they are masked by the simultaneous refrain. Therefore, it is not the semantic, but the phonemic and sonemic aspect of these songs that becomes more important, as well as assonances resulting from the polyphonic interaction of different texts. Musical rhythm is often based on the alternation of long and short syllables and is therefore closely linked to the rhythm of words. The opposition of long and short syllables is one of the more important elements of the Proto-Indo-European language, which is also preserved in Lithuanian. The long and short syllables form stable rhythmic patterns that are repeated throughout the song. This is particularly evident in the rhythmicity of asemantic refrains, where the rhythmic formulae of the words and the music often coincide, suggesting a kind of syncretism between music and language. This phenomenon is in some ways reminiscent of the concept of ‘musilanguage’ (Brown, 1999). Such overlaps between rhythmic formulas and the words they are assigned to can also be observed in Lithuanian polyphonic instrumental music, which is performed on *ragai* (wooden horns) or *skudučiai* (separate pipes). For example, the syncopated rhythmic formula ‘ti-ta-ti’ is conveyed by the word ‘Untyta’ (in the Lithuanian eastern dialect—‘A little duck’; LLIM; Paliulis, 1959, 66; 70–74; 104–105). The rhythm formula ‘ta-ti-ti-ta’ is remembered using the phrase *Griebk apačion!* (‘Grab downwards!’) which has no meaningful context and probably has only a mnemonic function (LLIM; Paliulis, 1959, 68). Overlaps between the text and the rhythm of the music may testify to the archaic origin of Lithuanian polyphonic songs. However, the stable rhythmic formulae and the coincidence of words and musical rhythm in Lithuanian polyphonic songs are not of a systematic nature and should not be considered a surrogate language, such as is known in the Yoruba ‘talking drum’ tradition of West Africa (Akinbo, 2021; Ros, 2021; Gonzįlez and Oludare, 2022). If such a surrogate language ever existed in the Lithuanian musical tradition, only vague traces of it have come down to our time.

## 5 Peculiarities of the musical language of *sutartinės* songs

Lithuanian polyphonic song *sutartinės* are characterized by a distinctive musical language, which is not typical of folk music of later origin. Their melodies are characteristic of archaic musical thinking: a narrow range, a limited number of notes in scale, syllabic rhythm (where one syllable of text corresponds to one note of the melody), archaic forms of polyphony (canon, antiphon, heterophony, paraphony), the frequent intervalic seconds between voices, polyrhythmia and polymodality, the syncretism of music, verbal text and movement. The following sections explain why these features of the musical language are considered archaic.

### 5.1 Features of *sutartinės* song melodies

When analysing the *sutartinės* songs, it can be noted that their melodies are usually within the range of a fourth (less often a fifth or sixth), and roughly correspond to the range of spoken language. The same range is used for the melodies of work songs, laments, children’s songs, and sung interludes from fairy tales, which belong to the most archaic folklore tradition. Carl Stumpf argues that ‘fourths or fifths are the largest intervals in many primitive^4^ songs <…> the entire ambitus of a song, the range from its lowest note to its highest, rarely exceeds this boundary’ (Stumpf, 2012, p. 71). The melodies of *sutartinė* are usually constructed from a limited number of scale steps—2–5 different notes (Čiurlionytė, 1969, p. 119). For narrow scales with a limited number of scale steps, the terms oligotony, oligotonic modes and the oligotinic system are used (Sliužinskas, 2003, p. 16, 26–27, 45).

**Figure 1:**
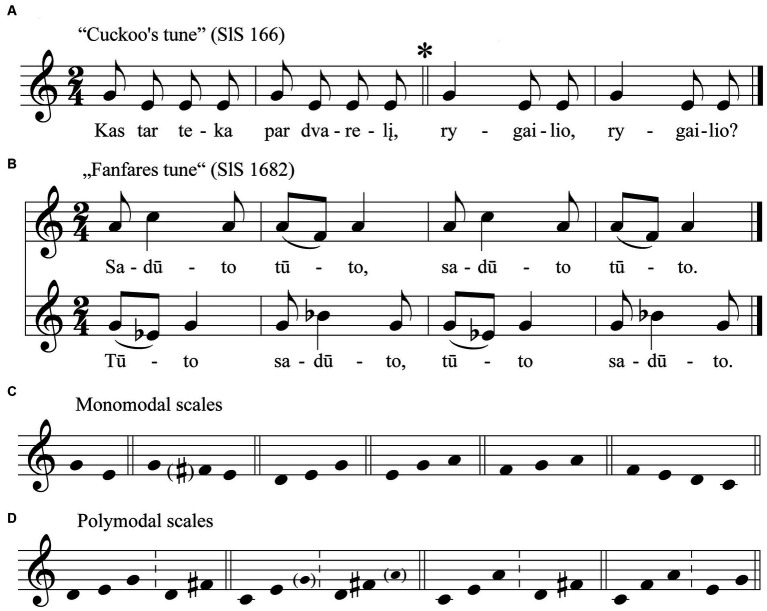
Scales typical of sutartinės melodies: **(A)** “Cockoo’s tune”; **(B)** “Fanfares tune”; **(C)** Monomodal scales; **(D)** Polymodal scales.

Bichord scales are the most elementary in terms of structure. In Bruno Nettl’s words, ‘[th]e simplest scales in the world comprise two tones. <…> The fact that they are distributed so widely and in areas isolated from each other has prompted speculation about their age; songs using them may be the oldest musical material surviving to this day’ (Nettl, 1956, p. 47, 48). Monomodal *sutartinės* based entirely on bichord are not abundant. Bichords *g-a* are extremely rare in *sutartinės* melodies (SLS; Slaviūnas, 1958/1959, 86, 1155), thought there are slightly more melodies based on the tertian bichord *g-e*, known as the ‘cuckoo’s tune’ (SlS; Slaviūnas, 1958/1959, 166, 194, 1203a; see Figure 1A). Songs based on anhemitonic trichords with various intervallic structures are more common: *d-e-g* (SlS; Slaviūnas, 1958/1959, 43, 828, 1667), *e-g-a* (SlS; Slaviūnas, 1958/1959, 126, 396, 604), *f-g-a* (SlS; Slaviūnas, 1958/1959, 90a, 186, 304, 591, 626, 846, 1161). Anhemitonic trichords are also characteristic of individual voices in mixed bimodal songs (SlS; Slaviūnas, 1958/1959, 116, 118). The other part of *sutartinės* is based on the hemitonic trichord, where the minor third is filled with a transitive tone, thus forming a ‘lament tune’ (SlS; Slaviūnas, 1958/1959, 290, 303a). By far the largest group of the songs is based on diatonic tetrachords: *c-d-e-f*, *d-e-f-g* and others (SlS; Slaviūnas, 1958/1959, 119a, 544).

The next largest group of songs, whose melodies are based on a “fanfare tune’ (Figure 1B), is characterised by its peculiarity. They are associated with instrumental pieces that are traditionally performed on traditional long woodwind trumpets, and are believed to be the result of a distinctive interaction between instrumental and vocal music (Paliulis, 1984, p. 87–95; Račiūnaitė-Vyčinienė, 2000, p. 158–173; Nakienė, 2001, p. 151). Most of these are bimodal, based on two keys separated by a second. A distinctive sound is characteristic of polymodal songs based on two major third bichords (these bichords consist of the 4th and 5th harmonics in the harmonic series). These scales have the unusual interval of an augmented fourth between the edge tones of these two bichords, which gives them a characteristic shading of the Lydian mode (SlS; Slaviūnas, 1958/1959, 483, 1442). Most of the pentachordal *sutartinės* are based on the bimodal ‘Fanfare tune’ as well. They are usually based on a bimodal structure consisting of the major triad *c-e-g* and the major third *d-f#* filling it (pvz., SlS; Slaviūnas, 1958/1959, 534, 547, 1439, 1443). There are also hexachordal polymodal songs in which the individual voices consisting of two major triads in different keys: *c-e-g* and *d-fis-a* [AM; Burkšaitienė and Krištopaitė, 1990, 35]. In the case of polymodal ‘fanfare’ songs, the intervals between voices are exclusively seconds. In the case of polymodal *sutartinės* with mixed scales, and for monomodal heterophonic *sutartinės*, the seconds alternate with other intervals (from prime to fourth). An extremely rare case is a monomodal unison *sutartinės* (SlS; Slaviūnas, 1958/1959, 544).

To sumarise: an analysis of the *sutartinės* melodies reveals that they are characterised by a wide variety of scales. *Sutartinės* can be monomodal (Figure 1C) or bimodal (with or without common tones; see Figure 1D). It is clear that most of their scales are of archaic origin. Anhemitonic bichords and trichords, hemitonic trichords and tetrachords, the ‘cuckoo tune’, the ‘lament tune’ and the ‘fanfare tune’—all of these scales belong to an archaic period of musical development; they predate the development of the diatonic octave scale.

Pure bimodal *sutartinės* (where the two modes do not share tones) are considered the original, most archaic form of those songs. Mixed bimodal (when two modes have at least one tone) and monomodal *sutartinės* (especially their unison variants) are of later origin. They have emerged over time as a result of the interaction between polyphonic and monophonic traditions and the simplification and degradation of polymodal songs. This trend of simplification, the ‘disappearance of *sutartinės’,* has been evident since the mid-20th century (Račiūnaitė-Vyčinienė, 2018, p. 401–404).

### 5.2 Characteristics of the *sutartinės* rhythm

**Figure 2:**
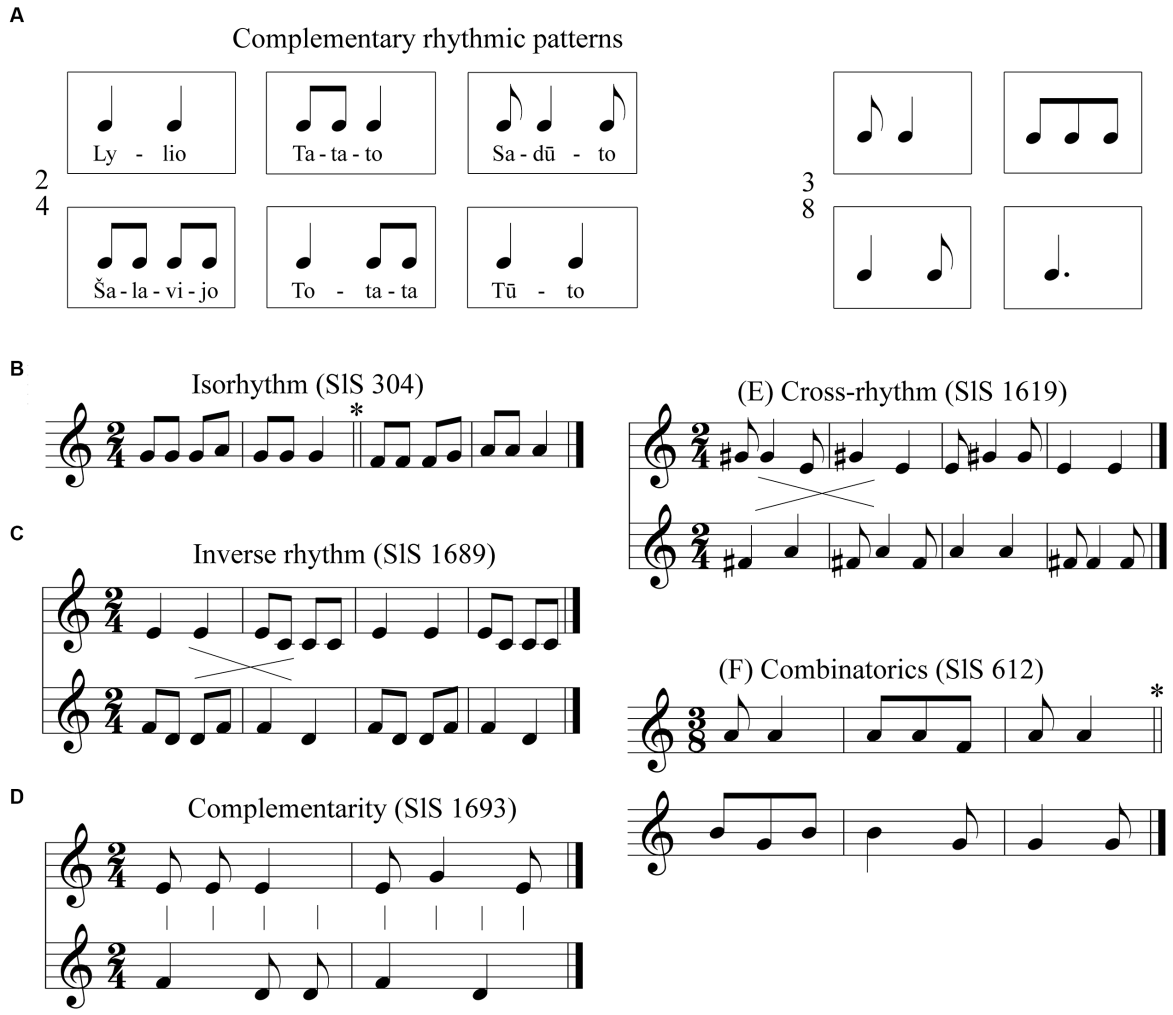
Examples of sutartinės rhythms: **(A)** Complementary rhytmic patterns; **(B)** Isorhythm; **(C)** Inverse rhythm; **(D)** Complementarity; **(E)** Cross-rhythm; **(F)** Combinatorics.

One of the most striking features of the *sutartines* is its distinctive polyrhythmic structure, full of syncopations and cross-rhythms. Rhythmic structures of the repertoire are characterized by syncretism—a relationship with the rhythm of words (the opposition of short and long syllables) and ritual movement. The rhythm of the *sutartinės* is essentially accentual, highlighting their symmetrical structure and their connection to archaic dance forms. About 90% of the *sutartinės* songs are in duple meters (2/4, 4/4, 4/8), the rest in triple or mixed meters (Slaviūnas, 1991, p. 18). The following schemes are commonly found in variable metre songs: 3/4 + 2/4 (SlS; Slaviūnas, 1958/1959, 1239, 1247, 1251), and 3/8 + 3/8 + 2/4 (SlS; Slaviūnas, 1958/1959, 116–118). There are also cases of polymetry, although these are quite rare (SlS; Slaviūnas, 1958/1959, 46, AM; Burkšaitienė and Krištopaitė, 1990, 17). *Sutartinės* are characterised by a motor rhythm with a clear metrical and syllabic structure (one note per syllable). However, the rhythmic formulae are quantitative rather than qualitative in nature: they are made up of long and short notes and are based on the opposition of duration. The long and short syllables form stable rhythmic formulae that are repeated throughout the song and often coincide with asemantic interjections that act as a refrain (see Figure 2A).

Thus, there is a clear correlation between the rhythmics of the poetic text and the musical rhythmic formulae. Often the rhythmic formulae of these refrain words and the music coincide: *tatato, ratilio* (∪ ∪ −), *totata* (− ∪ ∪), *sadūto, pijolka* (∪ − ∪), *tūto, lylio, lingo* (− −), *čibi ribi, kukal rože* (∪ ∪ ∪ ∪). The stable, constantly recurring rhythmic formulas of Lithuanian polyphonic songs fully correspond to the concept of C. Orff’s *Elementare musik* (Keller, 1954, p. 28), as they coincide with the rhythmic formations based on the natural rhythm of words. They testify to the early period of musical evolution preserved in this genre.

Polyrhythms are an essential feature of Lithuanian *sutartinės*. Examining the rhythmic interaction between the individual voices, the dominant principle of complementarity emerges. The rhythmic formulae of the individual voices are combined in such a way that a long note in one voice is filled with shorter notes in the other voice. The range of combinatorial possibilities in *sutartinės* extends from simple isorhythmic canons (SlS; Slaviūnas, 1958/1959, 304; see Figure 2B) to quite complex inverse (Figure 2C) and complementary (Figure 2D) rhythms. Analyzing the *foursomes* (four-part) *sutartinės* belonging to the more archaic layer, we can observe interesting examples of rhythmic inversion (SlS; Slaviūnas, 1958/1959, 1689, 1693), and cross-rhythm (SlS; Slaviūnas, 1958/1959, 1619; Figure 2E). The symmetry of the rhythm, the complementarity of the rhythm, is emphasized by the refrains of the lyrics. If one voice has the refrain *tatato* (∪ ∪ −), the other has *totata* (− ∪ ∪). If the first voice sings the refrain in *saduto tūto* (∪ − ∪ / – –), the second voice responds in *tūto sadūto* (− − / ∪ − ∪). The elementarity of the invariant rhythm formulae and their mutual complementarity guarantee their long-term stability.

There are also some quite interesting cases of rhythmic combinatorics. The rhythmic structure of the *sutartinė* ‘Tūto strazdeli’ (SlS; Slaviūnas, 1958/1959, 612) consists of three different rhythmic feet, which are arranged as follows: *a b a / b c c //*. When this song is performed in canon, all combinatorically possible variants of these foot pairs are created between the voices: *ab*, *bc* and *ac* (see Figure 2F).

### 5.3 Features of polyphony in *sutartinės* songs

The *sutartinės* are characterized by the variety of forms and processes of archaic polyphony. In their texture one can find practically all early polyphonic forms, from unison and antiphon, bourdon and paraphonia, to canon or free counterpoint (Slaviūnas, 2006, p. 24). Several intermediate forms of polyphony and their various derivatives are also common. Carl Stumpf rightly observes that there ‘are a variety of forms of polyphony, whose origins must go back a very long way. If our supposition about the origin of music is correct then this lies precisely in polyphonic (even if unconsciously polyphonic) singing or playing’ (Stumpf, 2012, p. 81). This is completely applicable in the case of Lithuanian polyphonic songs.

Singing in unison is not very characteristic of *sutartinės* songs. However, there are a few of threesomes *sutartinės* performed in the canon in which the first and second parts of the melody are identical but the texts differ (SlS; Slaviūnas, 1958/1959, 87, 112b, 500, 530, 544). There are also a few of four-part songs, where two pairs of voices alternately sing two different texts in unison (SLS; Slaviūnas, 1958/1959, 76, 112a, 312). It - is possible that unison polyphonic songs emerged as a result of the ‘wear and tear’ or degradation of traditional polyphony. Antiphon, or alternate singing in two groups—a transitional form between monophony and polyphony (SlS; Slaviūnas, 1958/1959, 17-15-1736) - is considered to be a precursor of polyphonic singing (Čiurlionytė, 1969, p. 257).

The heterophonic *sutartinės* represent an early form of polyphony (Slaviūnas, 2006, p. 24), a transitional, intermediate form of polyphony, with both monophonic and polyphonic features: they can be interpreted as imprecise unison. Neither polyphony nor monophony is adequately expressed in them (pvz., SlS; Slaviūnas, 1958/1959, 78, 98a, 154, 303a, 1263).

The usual ‘standing’ bourdon is almost absent in *sutartinės*. It occurs only in songs of more recent origin (SlS; Slaviūnas, 1958/1959, 1772, 1780, 1786). Račiūnaitė-Vyčinienė argues that the single-note bourdon in some of the voices in these songs is the result of instrumental musical influence and probably imitates a violin or bagpipe (Račiūnaitė-Vyčinienė, 2018, p. 219–236). The ‘walking’, i.e., variable-height bourdon is also quite rare (SlS; Slaviūnas, 1958/1959, 1771).

Paraphony (singing in parallel seconds) is characteristic of some twosomes (two-part) and foursomes (four-part) *sutartinės*. There are cases where two parallel melodies of the same rhythm are played at the same time, with an interval of a second between the voices at all times (SlS; Slaviūnas, 1958/1959, 1248). In some cases, the direction and rhythm of the melodies varies slightly, whilst maintaining the interval of a second (SlS; Slaviūnas, 1958/1959, 539).

The most popular, most frequently encountered form of polyphony in *sutartinės* songs is canon. All threesomes (three-part) *sutartinės* are performed as canon. However, only two voices are heard when they are performed, because the third part of the canon consists of pauses. A separate subgroup consists of threesomes *sutartinės* with antiphonal elements, where the voice leading the canon is supported by the other two alternating voices (SlS; Slaviūnas, 1958/1959, 125). There are also atypical three-voice *sutartinės*, where each voice sings different melody with different lyrics (SlS; Slaviūnas, 1958/1959, 97, 143).

The technique of contrasting polyphony is particularly common in foursomes and twosomes *sutartinės*. Singing counterpoint in seconds in terms of vocal performance is not an easy task in terms of vocal performance, and requires experience and skill in performing such songs. It is therefore acknowledged that the performance of such contrapuntal songs is quite complex and challenging (Račiūnaitė-Vyčinienė, 2009, p. 18). The counterpoint-based *sutartinės* are characterised by rhythmic inventiveness. They are full of syncopations, complementary and cross rhythms, with regular seconds between voices (SlS; Slaviūnas, 1958/1959, 1386, 1616, 1692 and others).

Thus, the *sutartinės* use the full range of polyphonic devices: from the canon in unison—through heterophony, paraphony in seconds and the canon—to the quite complex contrasting polyphony. This variety of polyphonic forms has developed over a long period of time. Some of these forms of polyphony (for example, alternate singing) are very archaic, perhaps tracing back to hunter-gatherer communities (or their descendants). In Lithuania, this variety of polyphony may have arisen from the interaction of different musical cultures: early polyphonic and later monophonic. It can be both the result of the development of the *sutartinės* from simple antiphonal songs towards a complex polyphony, and the consequence of the degradation and wear and tear (deterioration) of this polyphony.

Could this variety of polyphonic techniques have arisen from monophonic songs? Several different views exist on this point. There are indications that polyphony was an earlier form of music-making than monophony the latter therefore have arisen as a consequence of the degradation of polyphony. It can be assumed that the ancestors of mankind already performed polyphonic music while they had not yet developed sufficient rhythmic synchronisation and pitch-matching abilities to coordinate monophony. An example of such archaic polyphony that has reached our time is the vocal polyphony of the Aka, Baka and other pygmy ethnic groups in Central Africa. Curt Sachs argues: ‘Against our inherent misconceptions, today’s monophony is here and there in the oriental and primitive (see text footnote 4) world an end stage of what was polyphonics’ (Sachs, 1962, p. 176). J. Jordania takes a similar approach, presenting convincing scientific arguments (Jordania, 2022).

However, another approach is possible. Simha Arom’s careful analysis of examples of Central African polyphony and polyrhythmic music makes it clear that, far from being ‘primitive’, this music is based on complex polyphonic and polyrhythmic techniques (Arom, 1991). In the conclusion of his study devoted to Central African polyphony, Arom observes that these repertoires ‘display a striking contrast between their apparent complexity and the small number of elements from which they are constructed: a strict periodicity yielding cyclic forms, and <…> melodic structures, whose lengths are related in strictly proportional ratios’ (Arom, 1991, p. 658). It is obvious that this description would also be true to a large extent for traditional Lithuanian instrumental polyphonic music and a significant proportion of polyphonic songs *sutartinės*. It should be noted that some Pygmy and other ethnic groups (BaTwa Pygmies, Khwe Bushmen, El Molo, Hadza, and Sandawe) do not practise polyphonic music (Grauer, 2009, p. 422), so it cannot be excluded that polyphonic music may have developed later than the Stone Age. However, ethnomusicological studies do not provide sufficient evidence for a possible evolution of polyphonic music from monophonic. Simha Arom, who has studied descriptions of African music in reliable historical documents, notes that some techniques of polyphony and polyrhythmia were already in use at the end of the fifteenth century. ‘It is therefore likely that they are actually much older’ (Arom, 1991, p. 656). These discussions are an additional argument in support of the archaic origin of Lithuanian traditional polyphonic songs.

## 6 Spread of *sutartinės* in Lithuania and their analogues in the world

When studying the spread of this genre, it is evident that the *sutartinės* were not widespread in the whole territory of Lithuania, but only in the north-eastern part of Lithuania (Račiūnaitė-Vyčinienė, 2000, p. 46; Slaviūnas, 2006, p. 193). Some relics of second-based polyphony can also be found in neighbouring Latvia, in its south-eastern part. The area of distribution of the *sutartinės* is basically geographically identical to the territory inhabited by the ancient Baltic Selonian tribe between the Daugava and the Šventoji rivers. Therefore, some researchers believe that it is appropriate to associate the phenomenon of *sutartinės* with the legacy of the Selonians musical culture (Boiko, 1992; Račiūnaitė-Vyčinienė, 2000, p. 33, 47). This is due to the geographical features of the area. The area of distribution of these songs is in a lake-like, marshy area, away from navigable rivers. It is this geographical isolation that has helped this genre of songs to be preserved and to retain their archaic features. The ethnonym ‘sėliai’ is associated with the words *selėti* (‘to flow’), *sēliā* (‘island’, ‘hill’, ‘swamp’; Karaliūnas, 2015). The oldest Baltic settlements in the area date back to the 2nd millennium BC. Their most important trades were fishing and hunting. There are traces of the Selian language in Lithuanian and Latvian dialects, place names, hydronyms (Salakas, Zarasai, Sartai, Samavas, Sēlpils, Silene, Sīkumi). The Selonians became extinct in the 15th century when they were assimilated by other Baltic tribes. Assuming that the *sutartinės* are a heritage of the musical culture of the Selonians, their origins would have to be traced back to the time in which the Selonian tribe flourished.

For quite a long time, Lithuanian musicologists have argued that Lithuanian *sutartinės* are a unique isolated phenomenon with no analogues (Čiurlionytė, 1938, p. 271; Slaviūnas, 2006, p. 252). The fact that they have no analogues both among neighbouring nations and in the wider world, has also been considered by non-Lithuanian musicologists. Slovak musicologist Elschekova has argued that this type of polyphonic songs has no parallel (Elschekova, 1981, p. 240). Brambats thinks that it is difficult to find any genetic or historical context for Lithuanian *sutartinės* (Brambats, 1983, p. 26). However, more recent comparative studies have shown that the diaphony of seconds is universal in nature. Therefore, the diaphony of seconds characteristic of Lithuanian *sutartinės* is part of a broader phenomenon, known in German as *Schwebungs-Diaphonie* (Messner, 1980, 2013; Brandl, 1989).

According to some ethnomusicologists, the occurrences of diaphony in seconds in various cultures are of very ancient origin and is a relic of archaic musical cultures. Carl Stumpf argues that open dissonances without resolution between voices are typical in the music of primitive cultures: ‘Hence it is certainly fair to say that the roots of harmony are to be found in primitive peoples. They simply did not grow any further; harmony itself failed to materialise. Indeed, a primitive person does not find a major chord objectionable, though he does not long for one, certainly not for triads; and where he uses a dyad, our sense of hearing will typically perceive this as out of place. One finds open dissonances without resolution between vocals and accompaniment, or between instruments, in conspicuous places’ (Stumpf, 2012, p. 82). Polish ethnomusicologist Czekanowska argues that the polyphony of seconds is characteristic of some relic phenomena of Balkan, Indonesian and South African early music (Чекановска, 1983, p. 149). According to Brandl, the polyphony of seconds reflects a stage in human development when consonance had not yet been discovered (Brandl, 1989, p. 52).

Jordania, a researcher of traditional vocal polyphony from around the world, states that the Lithuanian *sutartinės* have almost no analogues in the world, and the closest analogues are the examples of the vocal polyphony of the indigenous Ainu people of northern Japan and the vocal polyphony of Nuristan in Afghanistan (Jordania, 2006, p. 155, 240–242, 246, 250). The Ainu of northern Japan are associated with the earliest Jōmon hunter-gatherer culture in Japan and are considered to be the inheritors of this culture (Savage et al., 2015); their culture existed in Japan from 10.000 to 300 BCE (Perri, 2016, p. 1,166–1,167). Lithuanian *sutartinės* and examples of Ainu vocal music are linked by the singing of the canon and the harmonies of seconds between voices. Ainu polyphonic songs, like Lithuanian *sutartinės*, are performed exclusively by women (Račiūnaitė-Vyčinienė, 2012, p. 196). It should be noted that the four types of Ainu polyphonic singing (canon, counterpoint, alternate singing and unison) are also characteristic of Lithuanian *sutartinės* (Kōchi, 2012, p. 104; Račiūnaitė-Vyčinienė, 2018, p. 278).

Lithuanian *sutartinės*, especially alternate songs, are typologically related to the Canadian Inuit vocal music genre *katajjait* (in Northern Canada) or *katajjaq* (in Greenland). This is a two-part vokal game performed by two women singing alternately. The sound is created by the use of asemantic syllables, sometimes resembling the voices of animals: deer, seals, or geese (Nattiez, 1999, p. 444; Nattiez, 1983, p. 457–476). There are also Lithuanian *sutartinės*, which imitate the voices of flying cranes (SlS; Slaviūnas, 1958/1959, 109a-b, AM; Burkšaitienė and Krištopaitė, 1990, 2, 3). These genres from distant cultures are linked by pairs of female singers, alternate singing (antiphon), and the ‘mouth to mouth’ singing manner (Nattiez, 1999, p. 401; Račiūnaitė-Vyčinienė, 2018, p. 312).

In European traditional music, the closest (most similar) examples of vocal polyphony can be found in some parts of the Balkans (Bulgaria, Bosnia and Herzegovina, Serbia, Albania). Ethnomusicologists studying this phenomenon tend to assume that it may be a relic of the musical culture of the Thracian-Illyrian tribes (Кауфман, 1966, p. 3–9). J. Jordania argues that bourdon polyphony, based on dissonances between voices, is a relic of Old European musical culture (Jordania, 2006, p. 222–223, 290). It is suggested that the focal points of Balkan peoples’ vocal polyphony are remnants of the old Balkan culture, a cultural tradition of the early Europeans (Rihtman, 1958, 1981, p. 81). Balkan vocal polyphony and Lithuanian *sutartinės* share the following common features: elements of polyphony, interweaving of voices, syllabic rhythm and second-based harmonies between voices (Elschekova, 1981, p. 240; Račiūnaitė-Vyčinienė, 2012, p. 107). Given this similarity between Lithuanian *sutartinės* songs and Balkan vocal polyphony, it can be assumed that these phenomena may date back to the Thracian-Illyrian-Baltic neighbouring period (i.e., around 2000 BC). A whole chapter of D. Račiūnaitė-Vyčinienė’s latest monograph is devoted to the parallels and possible connections between Lithuanian *sutartinės* and Balkan polyphonic songs (Račiūnaitė-Vyčinienė, 2018, p. 348–389).

The diaphony of seconds is also characteristic of music from much more distant cultures. Jaap Kunst noticed unexpected stylistic similarities in vocal music between the Balkans and the eastern part of the Indonesian island of Flores (Kunst, 1942; Kunst, 1953). Messner also found parallels with Papua New Guinean vocal polyphony. Examples of second-based polyphony have also been found on some Melanesian islands. According to P. Collaer, ‘[t]he parallels of major and minor seconds that appear in polyphonic voicing suggest certain proto-Malayan peculiarities’ (Collaer, 1965, p. 32).

These distant analogies show that the diaphony of seconds is a universal archaic phenomenon, locally preserved in various parts of the world. It shows traces of ancient substrate cultures. Therefore, Lithuanian musicology has already, albeit tentatively, raised the idea that the origins of the archaic polyphony of Lithuanian *sutartinės* should be sought in the culture of Old (pre-Indo-European) Europe^6^ (Račiūnaitė-Vyčinienė, 2018, p. 61, 109, 145, 384).

## 7 Conclusion

The archaic nature of the *sutartinės* genre is evidenced not by the separate elements of the musical language, but by the whole complex of them: (1) archaic forms of polyphony (antiphon, heterophony, paraphony, canon); (2) equivalence of separate voices in *sutartinė*; (3) characteristic second-based harmonies between voices; (4) narrow range of melodies; (5) limited number of different notes in melodies; (6) elementary melodic and rhythmic structures, opening up extensive combinatorial possibilities; (7) rhythmic combinatorics and complementarity, (8) syncretism of music, word and movement, (9) close links with instrumental music and dance (C. Sachs’s ‘The Magic Triad of Music’). However, there is nothing more fragile and ephemeral than a tradition passed on through oral transmission. Thus, the tradition of *sutartinės* itself has also changed and evolved, revealing the creativity of its successors and expanding the possibilities of this genre of songs. Only the very principles of archaic musical thinking have remained untouched by the passage of time.

Most of the *sutartinės* have a closed cyclic structure. They have no clear end and are therefore ‘infinite’ and ‘eternal’. Some of the *sutartinės* are performed with elementary choreographic movements. Each of the songs is like a ritual, a recurrence of an archetypal image. In this way, the performance of the *sutartines* ‘establishes’ space and time; in this sense, they are archaic and primal. All the voices of the *sutartinės* are equal and point to the egalitarian nature of the community of performers. Each individual voice in a *sutartinė* lacks the full meaning that the ensemble of all the voices creates. The performer of the songs has the experience of a ‘collective subject’. This way of making music is typical of archaic hunter-gatherer communities.

The analogues of *sutartinės* songs in world music are rare and geographically remote; their interplay is untraceable and considered as impossible. All these examples of archaic vocal polyphony are considered to be relics of ancient sub-stratum cultures and are associated with hunter-gatherer cultures. It is therefore reasonable to assume that the origins of the music of the *sutartinės* songs can be traced back to Old Europe, before the arrival of the Indo-Europeans (earlier than 3000 BCE).

A look into the world of *sutartinės* allows us to explore archaic ways of musical thinking, which are significantly different from the musical conventions with which we are familiar. The Lithuanian polyphonic songs *sutartinės* significantly enrich our knowledge of the evolution of the archaic vocal polyphony.

## Author contributions

EV: Writing – original draft.

## Footnotes

^1^http://www.sutartines.info/paliulis/result.php?lang=&p=127&tomas=1

^2^Source SlS is freely available on-line: http://www.sutartines.info/slaviunas/

^3^Source LLIM on-line: https://www.sutartines.info/paliulis/

^4^The term “primitive music,” used by Stumpf and other early comparative musicologists (Ellis, Hormbostel) as well as by later ethnomusicologists (Sachs, Nettl), is controversial and sounds somewhat anachronistic today. However, it must be pointed out that in the context of the scholarly literature of the time, it does not convey racist implications; quite the opposite: it was an early, imperfect attempt to question cultural racism in order to emphasise the cultural value of non-Western and ethnic musics [for further dicussion see: Savage (2022), p. 24].

^6^Archaeologist Maria Gimbutas has described Old Europe (before the arrival of the Indo-Europeans) as the ‘Mother Goddess’ era, dating it from 6500 to 3500 BCE. The epoch is characterised by a peaceful, matristic society (Gimbutas, 1982, 1989, 1991).
